# Transperineal laser ablation as treatment for benign prostatic obstruction: Safety, feasibility and functional outcomes—A pilot study

**DOI:** 10.1002/bco2.278

**Published:** 2023-08-24

**Authors:** Rob van Kollenburg, Luigi van Riel, Paul Bloemen, Theo de Reijke, Harrie Beerlage, Daniel de Bruin, Jorg Oddens

**Affiliations:** ^1^ Urology Amsterdam University Medical Centres Amsterdam The Netherlands; ^2^ Biomedical Engineering and Physics Amsterdam University Medical Centres Amsterdam The Netherlands

**Keywords:** benign prostatic hyperplasia, benign prostatic obstruction, laser ablation, lower urinary tract symptoms, minimal invasive treatment, transperineal laser ablation

## Abstract

**Background:**

Standard surgical treatment for lower urinary tract symptoms (LUTS) due to benign prostatic obstruction (BPO) requires anaesthesia and hospitalization. Transperineal laser ablation (TPLA) is a novel minimally invasive treatment for BPO, which has been performed using local anaesthetics and conscious sedation.

**Objectives:**

The aim of this study is to assess safety, feasibility and functional outcomes of TPLA for the treatment of LUTS in men fit also for standard surgery.

**Methods:**

This prospective, multicentre, interventional pilot study included 20 patients. Eligible patients were men ≥40 years of age, with urodynamically proven bladder outlet obstruction, a peak urinary flow of 5–15 mL/s and a prostate volume of 30–120 cc. All subjects underwent Soractelite™ TPLA using the Echolaser® X4 system. Two to four fibres were placed in the prostate, whereafter laser light induced coagulative necrosis. Twelve months of follow‐up included uroflowmetry, an ultrasound of the prostate and PROMs (IPSS and IIEF).

**Results:**

Twenty patients were treated with TPLA using local anaesthetics and optional sedation. Sixteen patients were treated in an outpatient setting, using only local anaesthetics in 12 of them; four were treated in the operating room, whereof two under general anaesthesia. No device related adverse events occurred, nor did any grade ≥3 adverse events during follow‐up. Post‐TPLA, 10 men continued spontaneous voiding, and 10 men developed a urinary retention treated by a temporary indwelling catheter for 15.2 ± 3.5 days. At 12 months, Qmax improved from 9.7 ± 3.5 to 14.9 ± 6.0 (*p* = 0.015), IPSS improved from 21.3 ± 5.2 to 10.9 ± 5.5 (*p* < 0.0001), QoL improved from 4.9 ± 0.9 to 1.9 ± 1.1 (*p* < 0.0001), IIEF‐15 total score remained stable and 11/13 patients (85%) preserved antegrade ejaculation.

**Conclusions:**

TPLA is a safe and feasible treatment for men with LUTS due to BPO. TPLA can be performed in an outpatient setting under only local anaesthetics. Functional and quality of life outcomes improved significantly at 12 months, and erectile function remained stable.

**Abbreviations**
BPO: benign prostatic obstruction
HGS: Haematuria Grading Scale
IIEF: International Index of Erectile Function
IPSS: International Prostate Symptom Score
LUTS: lower urinary tract symptoms
PROM: patient reported outcome measure; PVR, post‐void residua
TPLA: transperineal laser ablation
TURP: transurethral resection of the prostate
VAS: visual nalogue Scale

## 1 INTRODUCTION

Lower urinary tract symptoms (LUTS) are a significant burden in ageing men.^1^, ^2^ Benign prostatic obstruction (BPO) is a contributing factor to LUTS, showing an increasing incidence of up to 38 per 1000 person years at age 75–79.^3^ Almost all surgical therapies advised by the guidelines require general anaesthesia and hospitalization. In addition, the transurethral approach may cause complications such as haematuria and strictures.^4^, ^5^, ^6^ A minimally invasive and effective therapy is desired to minimize the patients' burden.

Soractelite™ transperineal laser ablation (TPLA) is a novel minimally invasive treatment that aims to induce local coagulative necrosis of prostate tissue. TPLA offers a transperineal approach that directly targets the prostate tissue, and by multifibre configuration approach, it enables extensive and individualized treatment areas. The treatment thus far is performed under local anaesthetics combined with conscious sedation in an operating or intervention room.^7^, ^8^ A study by Patelli et al. in men unfit for standard surgery showed safety and feasibility and promising outcomes for TPLA treatment.^7^

The aim of this study is to investigate the feasibility and safety of TPLA in healthy men fit for standard surgery. Furthermore, this study presents functional outcomes at 12 months of follow‐up. In addition, this study will describe the feasibility to perform TPLA procedures under local anaesthetics in an outpatient setting.

## 2 METHODS

### 2.1 Study design

This prospective, multicentre, interventional pilot study included 20 men. The study was approved by the institutional review board (registry number NL66057.018.18) and is registered at clinicaltrial.gov as ‘TPLA for BPO’ (NCT03653117). The study is in agreement with the IDEAL stage 2a recommendation.^9^ A comprehensive study protocol has been published previously.^10^

### 2.2 Study population

Participants were eligible if they were ≥40 years of age, had urodynamically proven bladder outlet obstruction, a Qmax of 5–15 mL/s, a prostate volume of 30–120 cc, spontaneous voiding, and were fit for standard transurethral desobstructive surgery. Exclusion criteria were previous prostate surgery, (clinical suspicion of) prostate cancer or a medical history with prostate or bladder cancer. All participants were recruited in the Amsterdam UMC, location AMC (Amsterdam, the Netherlands). Written informed consent was obtained.

### 2.3 Study procedure

**FIGURE 1:**
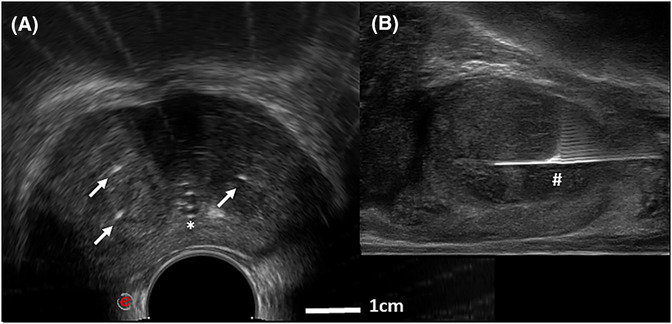
(A) Example of a transversal plane ultrasound image of the prostate. An indwelling catheter is placed for urethra identification (above the asterisk), and three needles are placed (arrows). (B) Sagittal plane view of the same prostate. The fibre is introduced into the needle, extending 1 cm from the needle tip (hash).

The TPLA procedure was performed in both the operating room as well as in the outpatient clinic. Local anaesthesia was performed by injection of the skin with Lidocaine 2%, 5–10 cc, and a periprostatic block with Lidocaine 2%, 20 cc. Conscious sedation (using 3 mg Midazolam intravenously) was optional at patients' preference. A single dose of ciprofloxacin 500 mg was administered as antibiotic prophylaxis, 1–2 h before the intervention. The patient was placed in lithotomy position, and a bladder catheter was introduced to visualize the urethra. The prostate was visualized using a bi‐plane rectal ultrasound probe (TRT‐33, MyLab Eight eXP, Esaote, Genoa, Italy). A needle guide system was used to support parallel needle placement (ABS33A and PLA33T adapter, Esaote, Genoa, Italy). Depending on the prostate volume, one or two fibres were placed transperineally in each of the two prostate lobes using ultrasound guidance, Figure 1. Thus, two to four fibres were used in total. Fibres were positioned parallel to the urethra with a minimum distance of 8 mm to the urethra and capsule, and 15 mm to the bladder neck, to protect these structures. The Echolaser Smart Interface device (Elesta S.p.A., Florence, Italy) was used to support needle positioning, in the majority of the procedures. The device assists in needle placement by safety margins projection on the ultrasound image.

The Soractelite™ TPLA treatment was performed using the Echolaser X4 system (Elesta S.p.A., Florence, Italy) with four separate continuous wave laser diodes operating at 1064 nm—a balance between blood and water absorption and tissue penetration. Fibres had a diameter of 300 μm and an NA of 0.22. Ablation was performed at 3 W power for 10 min, resulting in 1800 J per fibre. Depending on the prostate length, fibres could be 1 cm retracted, followed by a second ablation. Postoperatively, 8 mg of dexamethasone was administered and continued for 7 days to reduce oedema, and the bladder catheter was removed. A comprehensive overview of the study procedure has been described in an earlier published protocol paper.^10^

Following the procedure, patients were observed for several hours. Patients had to be able to void spontaneously before discharge. If a patient did not have spontaneous voiding and/or a residual volume of ≥500 mL, a Foley catheter was placed and a trial without catheter followed 10 to 14 days later. Urological medication was continued for 1 month and stopped at the first follow‐up check at 1 month.

### 2.4 Safety and feasibility

Safety was measured using the Clavien‐Dindo classification (version 5.0). The treatment was safe if no grade ≥3 adverse events occurred in the first 30 days following TPLA that were related to the treatment. Feasibility was measured by technically successful procedures without device‐related adverse events.

### 2.5 Patient data and follow‐up

Data were collected at inclusion and after 1, 3, 6 and 12 months of follow‐up. Collected data concerned medical history, adverse events, medication and physical examination on indication. Furthermore, Qmax was measured by uroflowmetry and post‐void residual (PVR) by abdominal ultrasound. Several patient‐reported outcome measures (PROMs) were used: International Prostate Symptom Score (IPSS) for voiding, International Index of Erectile Function 15 (IIEF‐15) for erectile function, Visual Analogue Scale (VAS) for pain, and Haematuria Grading Scale (HGS) for haematuria.^11^ Prostate volume was measured by transrectal ultrasound at baseline and 12 months of follow‐up.

### 2.6 Surgeons' experience

TPLA procedures were performed by two urologists who were experienced in transperineal approach of the prostate. Neither of the urologists had experience in TPLA procedures. Therefore, the two urologists were trained and supervised during the first three TPLA treatments by a medical doctor who was experienced in TPLA procedures.

### 2.7 Data analysis

Baseline characteristics, safety and feasibility were reported descriptively. Functional outcomes by Qmax, prostate volume and PROMs at all follow‐up moments were compared to baseline, using Wilcoxon signed‐rank test. Statistical tests were performed two‐sided, and *p* < 0.05 was considered significant. Statistics were performed and figures were created with GraphPad Prism for Windows (Version 9.1.0).

## 3 RESULTS

### 3.1 Baseline characteristics

The characteristics of included patients were spread well within the inclusion criteria. All baseline characteristics of the included patients can be found in Table 1.

**TABLE 1:** Patient characteristics at baseline.

	Mean ± SD (range)/No. of patients (%)
Age (years)	70.3 ± 7.3 (59–88)
Peak urinary flow (mL/s)	9.7 ± 3.5 (5–15)
Post‐void residual (mL)	61.8 ± 58.3 (0–212)
Prostate volume (mL)	65.5 ± 23.0 (31–117)
Median lobe present, *n* (%)	3 (15)
PSA (ng/mL)	5.0 ± 3.3 (0.9–13.5)
International Prostate Symptoms Score	21.3 ± 5.2 (12–28)
Quality of Life score	4.9 ± 0.9 (3–6)
IIEF‐15	35.4 ± 23.6 (5–70)
Urological medication:	
None	6 (30)
Alpha‐1‐blocker	12 (60)
5‐alpha‐reductase inhibitor	8 (40)
Beta‐3‐agonist	1 (5)

### 3.2 Procedure

The initial four procedures were performed in the operating room as the outpatient treatment room was not yet laser‐approved, and the following 16 procedures were performed in an outpatient clinical setting in a laser‐approved treatment room. The first two patients were treated under general anaesthesia by the preference of the anaesthesiologist. The following patients were treated using local anaesthetics, of which six patients opted for additional conscious sedation. Eight patients were treated with a single ablation using a fibre configuration of two (*n* = 3), three (*n* = 1) and four (*n* = 4) fibres. Twelve patients were treated with a consecutive second ablation after fibre pullback, using a fibre configuration of two (*n* = 4), three (*n* = 6) and four (*n* = 2) fibres (Table 2). One patient was treated with 3232 J instead of 3600 J as on one side the ablation was terminated earlier for safety reasons, due to prostatic capsule involvement impression at peri‐procedural ultrasound imaging. All procedures were performed successfully. Ten patients (50%) had spontaneous voiding following the TPLA procedure. Ten patients needed an indwelling catheter due to post‐procedural urinary retention. The catheter was successfully removed after 15.2 ± 3.5 days, range 10–20. One patient was admitted to the ward for observation because of bladder spasms caused by the bladder catheter.

**TABLE 2:** Perioperative and postoperative outcomes.

Location of treatment, no. of patients (%):	
Operating room	4 (20)
Outpatient clinic (laser‐suitable) intervention room	16 (80)
Anaesthesia used, no. of patients (%):	
Local anaesthetics	12 (60)
Local anaesthetics + conscious sedation	6 (30)
General anaesthesia	2 (10)
No. of laser fibres used, no. of patients (%):	
2 fibres	3 (15)
2 fibres with a pullback	4 (20)
3 fibres	1 (5)
3 fibres with a pullback	6 (30)
4 fibres	4 (20)
4 fibres with a pullback	2 (10)
Total energy delivered (Joules), mean ± SD (range)	8271 ± 3259 (3232–14 400)
Procedural time (minutes), mean ± SD, (range)	59 ± 14.0 (34–95)
Ablation time (minutes), mean ± SD (range)	17.2 ± 6.3 (10–27)
Post‐procedural spontaneous voiding, *n* (%)	10 (50)
Duration of hospitalization (hours), mean ± SD (range)	6.5 ± 5.0 (3–27)

### 3.3 Functional outcomes

**FIGURE 2:**
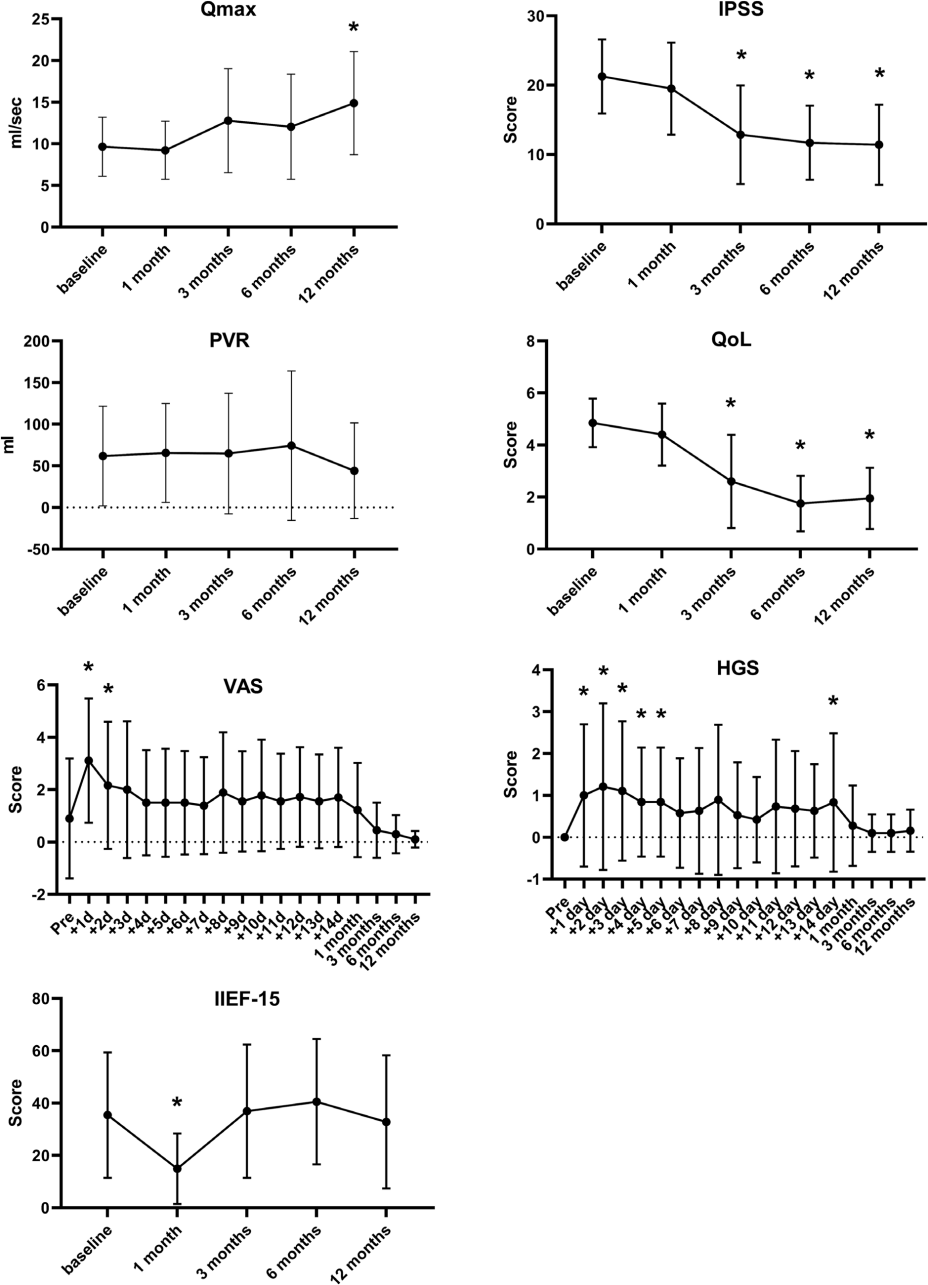
Functional outcomes, significant changes between baseline and any follow‐up moment are indicated by an asterisk. HGS, Haematuria Grading Scale; IIEF‐15, International Index of Erectile Function 15; IPSS, International Prostate Symptom Score; PVR, post void residual; Qmax, peak urinary flow; QoL, quality of life, VAS, Visual Analogue Scale for pain.

At 12 months, a significant Qmax improvement was observed of 5.2 ± 7.4 mL/s (*p* = 0.015). PVR did not change significantly with −10.8 ± 66.8 mL (*p* = 0.755). IPSS was reduced significantly by 9.7 ± 6.8 points (*p* < 0.0001), and QoL was improved by 2.9 ± 1.5 points (*p* < 0.0001) (Figure 2). Pain score and Haematuria Grading Scale score were significantly elevated in the first days following TPLA but were not significantly different between baseline and 12 months of follow‐up (Table 3). Prostate volume did not change significantly, −4.7 ± 18.1 mL (*p* = 0.251). Urological medication remained stopped in 17 patients at the end of the study. An alpha‐1‐blocker was started in two patients, 3 and 12 months following TPLA. Data of one patients were missing.

**TABLE 3:** Functional outcomes, all values show mean, SD, and (range).

	Baseline	3 months	6 months	12 months	Change (baseline–12 months)	*P* value (baseline–12 months)
Qmax (mL/s)	9.7 ± 3.5 (5–15)	12.8 ± 6.1 (4–25)	12.1 ± 6.1 (3–27)	14.9 ± 6.0 (4–29)	5.2 ± 7.4	**0.015**
PVR (mL)	61.8 ± 58.3 (0–212)	64.8 ± 70.4 (2–241)	74.2 ± 87.4 (0–330)	44.2 ± 55.8 (0–203)	−10.8 ± 66.8	0.755
IPSS	21.3 ± 5.2 (12–28)	12.8 ± 6.0 (3–29)	11.7 ± 5.2 (5–26)	10.9 ± 5.5 (4–24)	−9.7 ± 6.8	**<0.0001**
QoL	4.9 ± 0.9 (3–6)	2.6 ± 1.7 (0–6)	1.8 ± 1.0 (0–4)	1.9 ± 1.1 (0–4)	−2.9 ± 1.5	**<0.0001**
IIEF‐15	35.4 ± 23.4 (5–70)	36.9 ± 24.8 (5–69)	40.5 ± 23.3 (6–69)	31.1 ± 24.4 (7–70)	−2.8 ± 13.5	0.570
Prostate volume (mL)	65.5 ± 23.0 (31–117)	NA	NA	63.2 ± 20.6 (36–101)	−4.7 ± 18.1	0.251
VAS	0.9 ± 2.23 (0–8)	0.5 ± 1.0 (0–4)	0.3 ± 0.7 (0–3)	0.1 ± 0.3 (0–1)	−0.8 ± 2.4	0.313
HGS	0.0 (0)	0.1 ± 0.4 (0–2)	0.1 ± 0.4 (0–2)	0.2 ± 0.5 (0–2)	0.2 ± 0.5	0.50

*Note*: Bold emphasize the significant outcomes.

At 12 months of follow‐up, antegrade ejaculation was preserved in 11 out of 13 patients that had antegrade ejaculation at inclusion and available follow‐up data. The other two patients reported retrograde ejaculation at follow‐up. One patient reported retrograde ejaculation prior to TPLA and antegrade ejaculation during follow‐up, possible as a result of the alphablocker that was stopped during follow‐up. The other patients had either no ejaculatory function at inclusion, or missing follow‐up data. One patient reported erectile dysfunction following the TPLA procedure. IIEF‐15 total score dipped at the first follow‐up moment but returned to baseline at 12 months (Figure 2).

### 3.4 Adverse events

During the follow‐up, no related grade ≥ 3 adverse events occurred. Adverse events that occurred in the 30 days following TPLA are summarized in Table 4. Seven of 10 patients that needed an indwelling catheter following TPLA developed a symptomatic (urine culture proven) urinary tract infection after catheter removal and were treated by antibiotics without complications. Five patients experienced dysuria and three haematuria, all resolved after conservative treatment.

**TABLE 4:** Adverse events up to 30 days post‐TPLA.

Clavien‐Dindo	No. of patients with adverse events (%)
Grade 1	
Dysuria	5 (25)
Urgency	4 (20)
Haematuria	3 (15)
Pain	2 (10)
Frequency	1 (5)
Grade 2	
Urinary retention	10 (50)
Urinary tract infection	7 (35)

## 4 DISCUSSION

This study shows that TPLA is a safe and feasible option for men with LUTS due to urodynamically proven BPO who are fit for standard surgery. This study is the first to show that TPLA treatment can be performed using local anaesthesia only in an outpatient setting. Functional outcomes and quality of life by means of the Qmax, IPSS and QoL showed a significant improvement of 5.2 mL/s, 9.7 points and 2.9 points, respectively, at 12 months of follow‐up. Erectile function remained stable, and antegrade ejaculation was preserved in the majority of patients.

The results of this study are in line with outcomes of previous pilot studies. The significant Qmax, IPSS and QoL improvement of this study are in line with earlier studies by Patelli et al., Rienzo et al., Cai et al. and Frego et al.^7^, ^8^, ^12^, ^13^, ^14^ The setup of our study is grossly similar to the study of Frego et al. However, Frego et al. only report follow‐up data at 12 months of 45.5% of included patients. In our study, PROM and functional data at 12 months were available for 95% and 85% of patients, respectively. This makes the outcomes more reliable. Also, we only included patients with urodynamically confirmed bladder outlet obstruction. Thus improving the quality and robustness of our study results.

Tanneru et al. have compared several minimally invasive treatment for LUTS with TURP in a network meta‐analysis.^15^ The Qmax and PVR improvements found in our study are in line with the outcomes of prostatic urethral lift (PUL) and convective water vapour thermal therapy (CWVTT) as reported in a network meta‐analysis comparing several minimally invasive treatment for LUTS with TURP, at 12 months following treatment. The mean IPSS reduction of −9.7 at 12 months for TPLA is comparable to PUL and CWVTT as well. However, the QoL improvement of −2.9 points seems even better, as PUL and CWVTT resulted in a 2‐point reduction 12 months following treatment.

This study confirms the possibility to perform TPLA under local anaesthetics in an outpatient setting, as is in the meantime also shown in several other studies.^12^, ^16^, ^17^ This simplifies the room setup as continuous patient monitoring is no longer needed; the room only needs laser safety measures. In addition, all patients treated in an outpatient setting (*n* = 16) could be discharged within 6 h following TPLA, which included the trial to void. Compared to the first TPLA studies, this is a considerable total treatment time reduction.^7^, ^13^

And more importantly, this study is the first prospective trial that showed successful spontaneous voiding in half of the patients directly following TPLA. Factors that may have contributed within the group that continued spontaneous voiding could be a limited amount of energy (≤3600 J) or a high maximal detrusor pressure as measured during urodynamic investigation. These observations might serve as a basis for future studies' hypotheses on whether they can become predictive factors for decision‐making on indwelling catheter placement or voiding trial following TPLA. Especially because patients were hesitant to choose TPLA because of the risk of an indwelling catheter.

The number of patients that developed a urinary tract infection after indwelling catheter removal is substantially higher in this study when compared to earlier studies.^7^, ^8^ Three patients had an unsuccessful initial trial without catheter after 10–14 days, with indwelling catheter placement for another week. Catheter placement in a treated area with potential necrosis might have caused tissue damage and subsequent infection.

Preservation of sexual function is important to some patients. This study showed ejaculatory function preservation in 11/13 men that had antegrade ejaculation prior to TPLA treatment. One patient regained ejaculatory function following discontinuation of urological medication. These results are in line with earlier TPLA studies.^8^, ^13^ Thus, TPLA can be an interesting treatment for men that aim maximal chance of sexual function preservation.

A limitation of this study is the pilot design. The included 20 patients are sufficient to determine the primary objective: the safety and feasibility of TPLA in fit men. However, despite reporting of functional outcomes, the study was not powered for this objective. Furthermore, a learning curve was observed, resulting in a more extensive treatment with more often a fibre pullback in patients treated later on. This potentially led to better results throughout the study. Therefore, additional studies are needed with sufficient power for functional outcome evaluation. A randomized study comparing TPLA with TURP is being conducted.^18^ A recent abstract reported on the 3 years of follow‐up.^19^ Awaiting the full report on these data, more and longer follow‐up period is also desired for functional outcome evaluation, in line with other studies evaluating novel minimal invasive treatments for LUTS.^20^, ^21^

TPLA for BPO has been proven to be feasible and safe for application in men with LUTS. Functional outcomes increased significantly, up to 50% at 12 months. TPLA adds a new technique to the landscape of minimally invasive treatments for LUTS in men. This study showed that it can be performed in an outpatient setting under only local anaesthesia. The limited requirements for the treatment room, makes TPLA a treatment that does not seize on operating room time. However, randomized studies are needed to determine the position of TPLA in the landscape of minimal invasive treatments and standard LUTS surgery.

## AUTHOR CONTRIBUTIONS

Concept and design: RvK, DdB, TdR. Data acquisition: RvK, LvR, PB, TdR, JO. Data analysis and interpretation: RvK, DdB TdR, JO. Statistical analysis: RvK, JO. Drafting the manuscript: RvK. Critical revision of the manuscript for scientific and factual content: LvR, PB, DdB, TdR, HB, JO. Supervision: TdR, DdB, JO. Approval of the final manuscript: all authors.

## CONFLICT OF INTEREST STATEMENT

The authors declare that they have no competing interests.
